# Differential Impact of Social Frailty, Physical Frailty and Intrinsic Capacity Profiles on 2‐year Cognition in Healthy Older Persons: A Cluster Analysis

**DOI:** 10.1002/alz.092223

**Published:** 2025-01-03

**Authors:** Wee‐Shiong Lim, Cai‐Ning Tan, Audrey Yeo, Kalene Pek, Jia Qian Chia, Jun Pei Lim, Justin Chew

**Affiliations:** ^1^ Institute of Geriatrics and Active Aging, Tan Tock Seng Hospital, Singapore Singapore; ^2^ Institute of Geriatrics and Active Ageing, Tan Tock Seng Hospital, Singapore Singapore

## Abstract

**Background:**

Intrinsic capacity (IC) and frailty are inter‐related yet distinct constructs which encapsulate functional capacities of older adults. There is uncertainty how IC is related to and interacts with the social (SF) and physical (PF) dimensions of frailty to influence cognition. We aim to examine IC, PF and SF profiles and compare the association between identified subtypes with change in cognition at 2 years

**Methods:**

We studied 230 healthy community‐dwelling older adults (age:67.2±7.4 years) from the GeriLABS‐2 cohort study. We assessed: 1) IC, using modified Integrated Care for Older Adults (ICOPE); 2) PF, using Fried Frailty Phenotype; 3) SF, via 8‐item Social Frailty Scale. To identify subtypes, we performed two‐step cluster analysis with age, PF, SF, IC and CFAB. Cluster associations with change in cognition [Chinese Mini Mental State Examination (CMMSE, range:0‐28) and Chinese Frontal Assessment Battery (CFAB, range:0‐18)] at 2 years were examined using hierarchical linear regression sequentially adjusted for age, gender and education (step 1); PF, SF or IC (step 2); and PF, SF and IC (step 3).

**Results:**

Three distinct clusters were identified – C1: PF/High IC (N = 54, 24%); C2: SF/Low IC (N = 69, 30%); and C3: Robust (Physical/Social) /High IC (N = 106, 46%). There were more men in C1, whilst C2 was older with fewer years of education and scored lowest in CMMSE [C1 to C3, mean (SD): 26.20(1.58) vs 25.32(1.86) vs 26.60(1.53), p<0.001] and CFAB [17.20(0.86) vs 14.59(2.28) vs 17.26(1.04), p<0.001]. At 2 years, C1 experienced greatest improvement in CMMSE [0.69(1.60) vs 0.11(1.81) vs 0.03(1.70), p = 0.088) vis‐à‐vis C2 for CFAB [0.06(1.22) vs 1.21(2.07) vs ‐0.19(1.64), p<0.001). The association of CMMSE improvement in C1 became non‐significant when adjusted for PF (β = 0.63, P = 0.261), SF (β = 0.60, P = 0.058), or IC (β = 0.57, P = 0.072), but remained significant for CFAB improvement in C2 even in step 3 (β = 1.423, P = 0.004).

**Conclusion:**

Amongst healthy community‐dwelling older adults, SF and IC are complementary to physical frailty measures for better profiling of at‐risk individuals with lower executive function beyond general cognition. Further research is required to explicate the differential longitudinal improvement between clusters in general cognition and executive function and the factors underpinning the improvement.

